# MeLa: A Programming Language for a New Multidisciplinary Oceanographic Float

**DOI:** 10.3390/s20216081

**Published:** 2020-10-26

**Authors:** Sébastien Bonnieux, Dorian Cazau, Sébastien Mosser, Mireille Blay-Fornarino, Yann Hello, Guust Nolet

**Affiliations:** 1 Université Côte d’Azur, Observatoire de la Côte d’Azur, CNRS, IRD, Géoazur, 06560 Valbonne, France; hello@geoazur.unice.fr (Y.H.); nolet@princeton.edu (G.N.); 2 Université Côte d’Azur, CNRS, I3S, 06900 Valbonne, France; bblay@unice.fr; 3 Lab-STICC, UMR 6285, CNRS, ENSTA Bretagne, 29238 Brest, France; dorian.cazau@ensta-bretagne.fr; 4 Département d’Informatique, Université du Québec à Montréal, Montréal, QC H3C3P8, Canada; mosser.sebastien@uqam.ca; 5 Department of Geosciences, Princeton University, Princeton, NJ 08544, USA

**Keywords:** acoustic monitoring, oceanography, Model Driven Engineering, Model Based Programming, Domain Specific Language, embedded system, embedded software, Digital Signal Processing

## Abstract

At 2000 m depth in the oceans, one can hear biological, seismological, meteorological, and anthropogenic activity. Acoustic monitoring of the oceans at a global scale and over long periods of time could bring important information for various sciences. The Argo project monitors the physical properties of the oceans with autonomous floats, some of which are also equipped with a hydrophone. These have a limited transmission bandwidth requiring acoustic data to be processed on board. However, developing signal processing algorithms for these instruments requires one to be an expert in embedded software. To reduce the need of such expertise, we have developed a programming language, called MeLa. The language hides several aspects of embedded software with specialized programming concepts. It uses models to compute energy consumption, processor usage, and data transmission costs early during the development of applications; this helps to choose a strategy of data processing that has a minimum impact on performances. Simulations on a computer allow for verifying the performance of the algorithms before their deployment on the instrument. We have implemented a seismic P wave detection and a blue whales D call detection algorithm with the MeLa language to show its capabilities. These are the first efforts toward multidisciplinary monitoring of the oceans, which can extend beyond acoustic applications.

## 1. Introduction

### 1.1. Context

Scientists all over the globe are permanently monitoring how our planet is changing. Knowing how much heat is stored in the ocean, how fast the sea levels are rising, and sea ice is melting, where living ecosystems are migrating in response to anthropic activity, are only a very few of the many essential questions to understanding the current state and changes in the ocean and climate. This information is critical for assessing and confronting oceanic and atmospheric changes that are associated with global warming and they can be used by decision-makers, environmental agencies, the general public, and in measuring our responses to environmental directives.

Oceans have been monitored since the 19th century [1]. The first oceanographic campaigns were done from ships with manually handled instruments. When electronics and batteries were emerging, instruments started to become autonomous [2]. For moored instruments, like moored lines [3,4] or Ocean Bottom Seismometers (OBS) [5], they can now be deployed at sea for periods up to several months or years. However, the elevated costs of maintenance reduce our ability to deploy them globally. Alternatively, remote sensing based on satellites [6] allows for working at a global scale, but only has access to the ocean’s surface and has relatively low spatial and temporal resolutions in comparison to *in-situ* sensors.

Nevertheless, satellite communication systems provide the necessary technology to locate and transmit in near real-time data that were collected by autonomous underwater vehicles. Such vehicles include profiling floats [7,8] and wave gliders [9]. Both have different advantages and drawbacks, depending on the usage. Profiling floats are widely used in the Argo (https://argo.ucsd.edu/about/) program with thousands of floats deployed world-wide [10]. They monitor the temperature and salinity from the surface to a depth of 2000 m to study the climate.

Most of the floats follow the same operational cycle: (1) they descent to a depth programmed by the operator, (2) they park at this depth during several days or weeks and drift with currents, (3) they ascent to the surface, and (4) they measure their position with a Global Positioning System (GPS) receiver and send their data through a satellite link. One operational cycle is called a dive. The depth is regulated by changing the float density using an external bladder that was filled with oil. Measurements are done during any step of the dive by sensors integrated into the float to measure conductivity, temperature, depth, chlorophyll, nitrate, as well as acoustic signals and others.

In more recent works, Underwater Passive Acoustic (UPA) measurements have been integrated into profiling floats for different monitoring applications, such as whale tracking in marine ecology [11] and above-surface wind speed or rainfall estimations in marine meteorology [12,13]. In this field, a breakthrough has recently been obtained by seismologists with the Mobile Earthquake Recording Device in Marine Areas by Independent Divers (Mermaid), an autonomous float equipped with a hydrophone and a smart acquisition board able to recognize seismic sounds [14]. The recognition of seismic sounds allows it 1) to trigger the ascent of the float in order to obtain a precise estimation of the recording position and 2) to transmit only relevant seismogram data through the low bandwidth satellite link. So far, 60 floats have been deployed to image mantle plumes beneath hotspots in the Pacific Ocean.

### 1.2. Motivations and Objectives

In this paper, we aim to develop a multidisciplinary version of the Mermaid float, making it possible to combine different monitoring applications during the same campaign. Although we focus on UPA monitoring, the float is not limited to acoustic and can integrate other sensors.

The main motivation of our work is to enable scientists to write signal processing applications for the instrument. Indeed the sensors, and more especially the acoustic, generates high volume of data (e.g., a five minutes recording at 78.1 kHz produces 70 MB of data, and 7 TB for one year). These data can be stored by the floats, but, due to cost effectiveness, the floats are usually not recovered from the oceans, as it is the case with most Argo floats. The satellite communication system has a very limited bandwidth and it is not capable of transmitting such an amount of data. Moreover, many applications such as monitoring of earthquake or volcanic activity, require data transmission in (quasi) real time. An algorithm that is generic enough to handle different signal processing applications from different domains does not exist. Even machine learning algorithms have different architectures, depending on the application. Thus, the Mermaid cannot be configured with just a few parameters, it must be programmed with fully fledged applications.

However, developing signal processing applications to be embedded in an instrument such as Mermaid is challenging for the following four reasons:

1. Embedded software programming requires specific technical skills, and it can be off-putting for less technically skilled scientists who will have to learn C language programming and know specialized technical details regarding the instrument, such as the operation of the real time operating system, micro-controller, sensors, etc.
2. The embedded applications must comply with the limited resources of the instrument. Otherwise they may not behave as expected, induce elevated costs of data transmission, or considerably reduce the instrument life time.
3. The embedded applications must be reliable, without software bugs, and efficient, with a minimum impact on the instrument resources. Any miss-conceived code may compromise the instrument that is not directly accessible when deployed in the oceans. Less technically skilled developers are more prone to writing miss-conceived code.
4. The embedded applications developed independently and installed on the same instrument must not interfere with each other. Whether the applications are installed alone or with other applications their behavior must not change.

To overcome these challenges, we have developed a programming language dedicated to the Mermaid. This language is designed in order to meet the needs of signal processing experts and it does not require embedded software programming skills. The language is called MeLa, for Mermaid Language, and it is presented in the next section.

## 2. A Programming Language Based on Models

### 2.1. Models for Programming

Scientists use models to understand the world, for example, with climate models. Engineers use models to develop new systems (i.e., a bridge, a computer program). These models are specific to a domain of expertise, for example, electronic engineers use models of resistances or transistors. The models are assembled together to develop a system, for example, an electronic circuit. A language, graphical or textual, which allows for editing the models, is usually called a Domain Specific Language (DSL) [15]. The MeLa language is a DSL dedicated to the development of signal processing algorithms for the Mermaid floats. Models can be used in several ways in order to respond to the challenges that are introduced in the first section.

First, models allow for us to represent systems at several levels of abstraction. In software engineering assembly instructions are low-level models, whereas functions or tasks of an operating system are models with a high-level of abstraction. The MeLa language gains in abstraction with models that are dedicated to the development of applications for the instrument. Instead of programming applications with tasks, which need an expert in embedded software, the MeLa language offers models called acquisition modes. Using these models, the developer does not have to manage the execution priority of tasks, or the synchronization of execution with other tasks. Instead, developing an application with an acquisition mode only requires that the user defines the input sensor and the sampling frequency. This high level of abstraction allows for developers that are not embedded software experts to write applications for the instrument (challenge 1).

Second, models can be used to compute properties of the developed system before building it. The models of the MeLa language allow estimating properties of the developed applications, such as the lifetime of batteries, the cost of satellite transmission, and the processor usage, but other properties can also be incorporated in the models. Because the models are associated with the programming language, the estimations can be linked to the content of the applications. For example, the estimations can indicate which function uses most of the processor time. Thus, using models allows verifying that the instrument limits are not exceeded (challenge 2). Moreover, these estimations are computed during the writing of the applications, improving productivity. This would not be the case if measurements were done on a real instrument with specialized equipment; it would require expertise and time to realize the measures and interpret them.

Third, models are used to generate the specific embedded software specific code to program the instrument. The code generation process is managed by a tool that is integrated in the language, such as a compiler. The transformation rules to generate the code are defined by embedded software experts; this ensures having a reliable and efficient code (challenge 3). For example, the acquisition modes generate several tasks, with their synchronization mechanism and execution priority. The embedded software code would not be reliable and efficient if directly written by a non-expert. It is also possible to generate several specific codes to program different platforms. In our case, we also generate code to execute the applications on a personal computer, it allows for scientists to settle the applications and verify that they behave as expected.

Finally, models allow for composing (i.e., combine) applications that have been developed independently (challenge 4). The composition of applications consists mostly of verifying that applications are compatible in terms of both used computational resources (e.g., they cannot use more resources than what is available) and sensors (e.g., they cannot share a sensor if their configurations on this sensor differ). The concurrent execution of applications is also managed by the operating system at the time of execution time.

**Figure 1:**
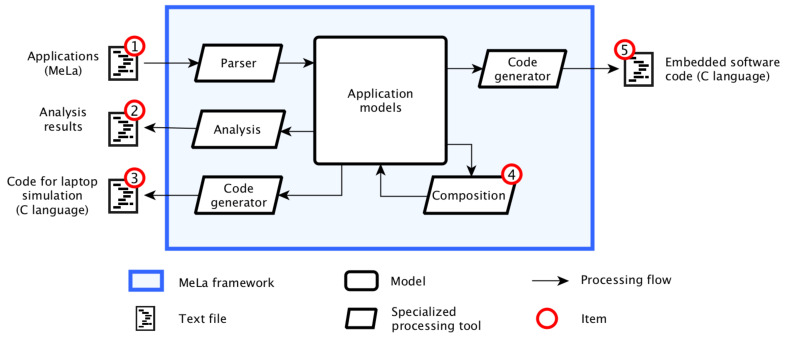
Models used in MeLa.

Figure 1 illustrates how models are used in MeLa:

1. Scientists write applications in MeLa, which avoids embedded software concerns. The applications written in a text file are transformed into models (implemented as Java objects) with a parser.
2. The analysis verifies that the limits of the floats are not exceeded and the results are returned to developers allowing them identify problems and correct them.
3. The code for simulation on a computer is generated to settle the applications and verify that they behave as expected.
4. Composition combines several applications to install on the same float after verifying that they are not incompatible.
5. The embedded software code to program the float is generated.

More details about the use of models in the MeLa language can be found in [16].

### 2.2. Description of MeLa

The MeLa language is both imperative and declarative. The imperative part of the language allows for writing the content of the algorithms with sequences of instructions, conditions, and loops. The declarative part allows for declaring the depth and duration of the float dives, when to execute an application, and which sensor an application has to use. The MeLa language is implemented with ANTLR [17], a DSL that is dedicated to the creation of other languages. The models behind MeLa are written in Java, an object-oriented programming language. The code to program the instrument generated from the models is in C language.

**Figure 2:**
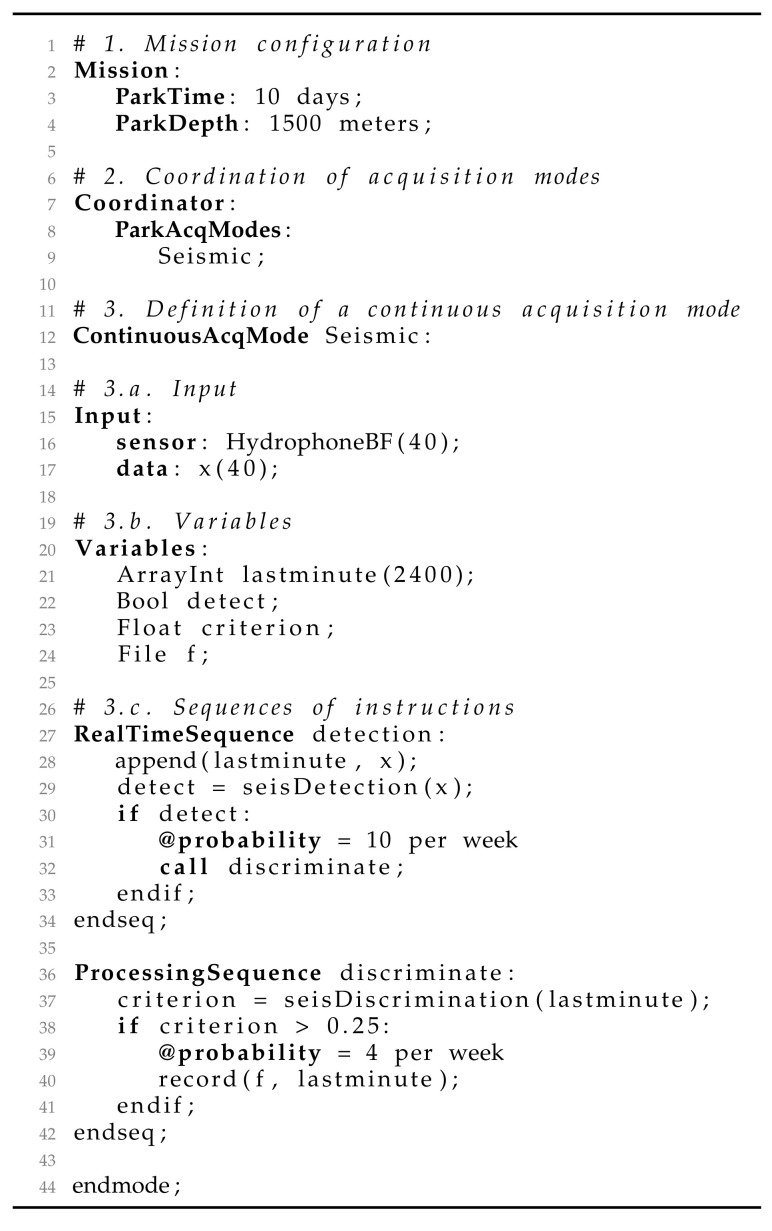
Application example. The functions ’seisDetection’ and ’seisDiscrimination’ have been left to keep the example short, they do not exist in the language, the real algorithm for seismic detection is presented in Section 4.1.

An example of an application written with MeLa is given in Figure 2. This is a very simplified version of a seismic detection application. The mission configuration part (lines 1–4) allows for the developer to define the depth and duration of a dive. The coordinator (lines 7–9) allows her to define when to execute an algorithm during the descent, parking or ascent steps of the dive. In this example, she has only chosen to run the Seismic algorithm during the parking stage, because the ascent and descent are too noisy for seismic monitoring.

The algorithmic part of an application is contained in what we called acquisition modes. The developer has to define the input of the acquisition mode. There are two parameters for the input, 1) the sensor with its sampling frequency and 2) the array name and size in which the sensor puts the samples. In the example, the sensor is the low frequency hydrophone with a sampling frequency of 40 Hz (line 16). The samples are put in an array, called x, containing 40 samples (line 17). The samples are processed when the array is fully filled by the sensor.

**Figure 3:**
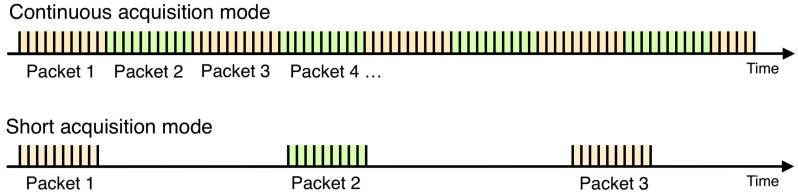
Data packets for ContinuousAcqMode and ShortAcqMode.

There are two kinds of acquisition modes, the ContinuousAcqMode, for which packets of data are continuously recovered, in a streamed way, and the ShortAcqMode for which single packet of data is recovered. The latter can be periodically executed with a time interval defined in the coordinator (e.g., ParkAcqModes: Temperature every 1 h). Figure 3 provides a comparison between the two acquisition modes. Choosing an acquisition mode mainly depends on what is monitored and it has implications for the resources that are used by the applications. Details about how to choose an acquisition mode are given in Section 3.

The algorithmic part inside a continuous acquisition mode is executed periodically, each time that the sensor sends a packet of data. During the acquisition, it is necessary to ensure that all of the packets of data are processed, in order to guarantee the integrity of acoustic signals. On the other side, it may be necessary to suspend the acquisition to temporary execute an algorithm that has a long execution time. The MeLa language separates the content of acquisition modes in two kinds of sequences of instructions in order to help the developer to think about these important aspects:

1. The RealTimeSequence (lines 27–34) is associated with real time constraints to guarantee that all the data from the sensor are processed. The execution time of the sequence must be shorter than the time between each packet of data, and other applications cannot delay the processing of a block of data until the point of missing a packet of data. The method used in order to verify these constraints is a scheduling analysis and it is described in Section 2.5.1.
2. The ProcessingSequence (lines 36–42) does not have real time constraints. Using this sequence means that the developer accepts to miss some data from the sensor. However, this sequence allows for calling instructions with a long execution time that would raise an error inside a RealTimeSequence.

A RealTimeSequence can only be called from a ContinuousAcqMode. Indeed, the acquisition is stopped between each execution of a ShortAcqMode, thus it does not require a RealTimeSequence. It is also verified that the execution time of a ShortAcqMode is less than its execution time interval (the time between two subsequent calls). However, a delayed acquisition in this case is less problematic, since it would not affect the integrity of data (the acquisition is suspended anyway). Furthermore, it is very unlikely, because the execution time interval of this mode is set in the coordinator and it is intended to be of several minutes or hours (not less than one minute).

Inside the sequences of instructions, the developer writes the algorithm with variables and functions. The variables must be first declared inside each acquisition mode in a section, called Variables (lines 20–24). The data types that are currently available to declare a variable are given in Table A1. The functions are called inside the sequences of instruction (lines 28, 29, 37 and 40 of Table A2). A list of functions currently available is given in Table A2. Many of them have been selected from the CMSIS DSP library ( https://www.keil.com/pack/doc/CMSIS/DSP/html/index.html).

The functions can be organized using statements that are commonly found in imperative programming languages, such as if conditions and for loops. A particular aspect of the if statements in MeLa is that each branch requires a probability to be given for its occurrence (e.g., the probability that an earthquake signal is strong enough to trigger a detection). That probability is used to compute properties of the application, especially the energy consumption and the volume of data transmitted through satellite communication (see Section 2.4.2).

#### 2.3. Mermaid Float Architecture

**Figure 4:**
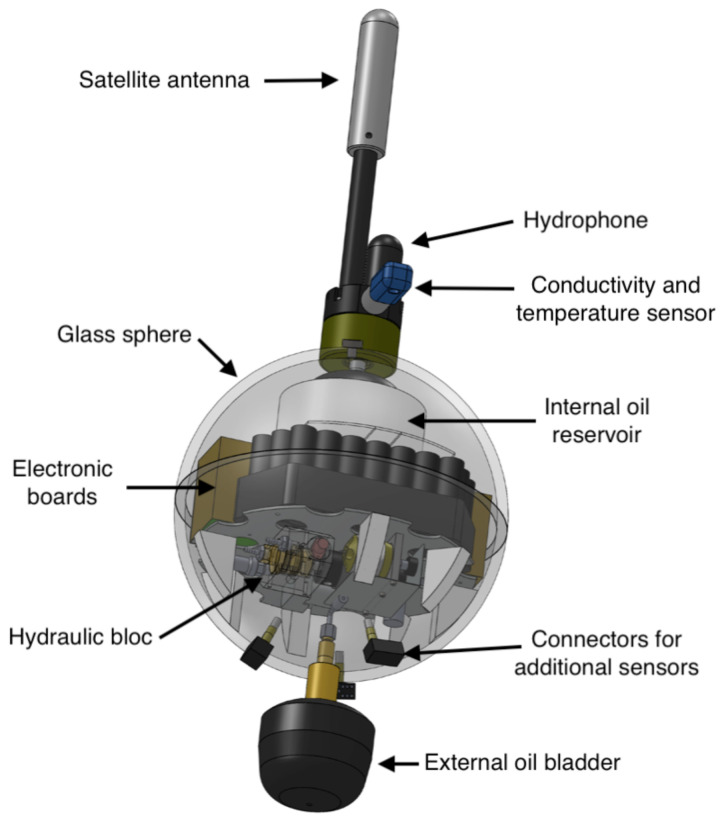
Mermaid float.

The instrument (Figure 4) is made of a glass sphere that resists until a depth of 5000 m. A hydraulic circuit transfers oil between a tank inside the sphere and an outside bladder. When the bladder deflates, the instrument volume decreases and its density increases, allowing it to dive. Up to eight sensors can be installed on the instrument, although currently we have only experimented with a hydrophone and a Conductivity Temperature Depth (CTD) sensor to monitor water temperature, salinity and density. The hydrophone has two outputs to monitor sounds at low and high frequencies, between 0.1 Hz to 100 Hz and between 10 Hz to 10 kHz. The sampling frequency of each output can be chosen by the developer. A satellite antenna at the top of the instrument is used for positioning with the GPS and data transmission with the Iridium Router-Based Unrestricted Digital Internetworking Connectivity Solutions (RUDICS) protocol. Batteries with a total capacity of 4 kW.h, equivalent to four hundred times those of a smartphone (~10 W.h), power the float for years or months, depending on the power consumption of the applications. For the Mermaid floats currently operating to monitor the seismic activity, the expected lifetime is five years.

The float contains two electronic boards. The pilot board manages the hydraulics (i.e., depth regulation) and communications (i.e., GPS, and Iridium). The acquisition board manages the sensors and processing of data. It has 512 kB of programmable memory, 8 MB of Static Random Access Memory (SRAM) and 128 GB of flash (SD card). The microcontroller is based on a Cortex-M4 core that integrates a Digital Signal Processor (DSP) and works at a frequency of 32 MHz. This is a very limited configuration when compared to a smart phone, but it has a low power consumption that is adapted for long-term operation.

Both electronic boards are programmed with C languagel which allows writing software with efficient execution time and low memory usage. Both have a Real Time Operating System (RTOS) allowing them to execute several tasks concurrently. The pilot board software can be configured with several parameters, such as the dive duration, depth, and other ones more technical, such as the time interval between each depth correction. The acquisition board has access to the sensors and can be programmed by scientists with MeLa, which generates C code. Both of the boards can communicate with each other, for example, the acquisition board can ask to the pilot board for the ascent of the float.

#### 2.4. Code Generation

##### 2.4.1. Overview

**Figure 5:**
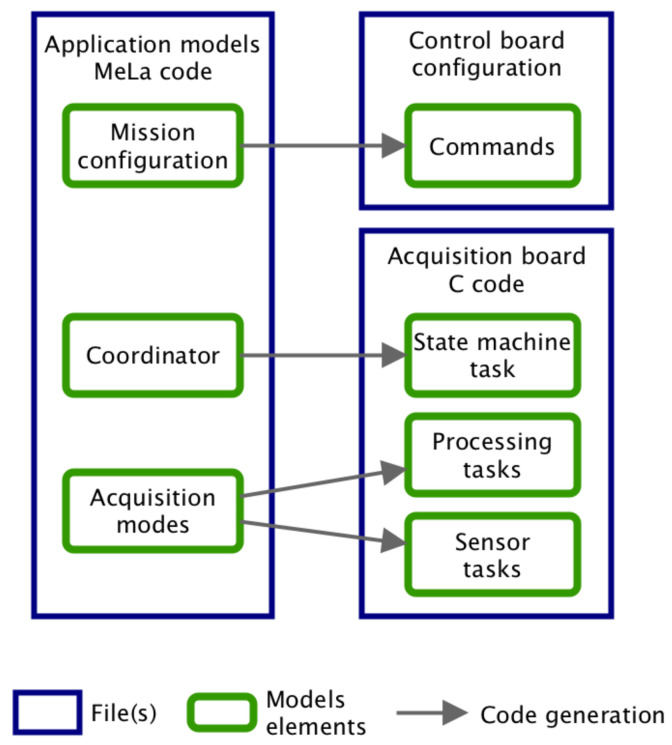
Code generation mapping.

The applications written in MeLa are transformed in C code that is suitable to program the Mermaid floats. This process is called code generation and it is equivalent to a compiler, but it generates C code instead of the binary file used to program the microcontroller. The mapping between the MeLa code and the generated code is illustrated in Figure 5.

A file to configure the pilot board is generated from the mission configuration part of the MeLa code. The rest of the MeLa code is used to generate the C code to program the acquisition board. The coordinator is mapped to a task containing a state machine (i.e., a model of computation) [18] that manages the execution of acquisition modes and the messages exchanged with the pilot board. The acquisition modes are converted to processing tasks that contain the sequences of instructions and sensor tasks handling the data from sensors (one sensor task can feed several processing tasks).

##### 2.4.2. Priority Rules

The real time operating system requires a priority of execution to be defined for each task. The highest priority is assigned to tasks with the shortest time interval between each execution (i.e., the shortest period of execution), which is a rate-monotonic priority assignment [19]. An exception is during the execution of a processing sequence of a continuous acquisition mode; the lowest priority is assigned to the processing sequence that can have a long execution time, so that the other tasks cannot be blocked.

##### 2.4.3. Benefits of MeLa for Embedded Software Programming

In MeLa, the functions accept different data types. For example, the max() function that searches the maximum value and index in an array accept integer and floating point arrays. On the contrary, the C language requires specific functions for each data type, for example, maxArrayFloat() and maxArrayInt(). In the MeLa library, the functions are defined by their MeLa name, their parameters types, and their C name. Several functions can have the same MeLa name, but different C names with different parameters types. When a function is called from the MeLa language, the parameter types are used to choose the function with the appropriate C name. For example, if the MeLa code is max(a) with a an array of integers, the corresponding function is the one that accepts arrays of integers and have the C name maxArrayInt(), if a is an array of floats the corresponding function is the one with the C name maxArrayFloat(). This mechanism is also called type inference, it makes programs easier to read and write by reducing the language verbosity [20].

The verbosity is also reduced for variable declarations. A variable declared in MeLa can have a much more verbose C equivalent. For example, declaring a StaLta variable in MeLa requires only one line, StaLtaFloat stalta(5, 15, 10), but declaring it in C requires three lines, because it corresponds to an array, a circular buffer structure that contains the array, and a *stalta* structure that contains the circular buffer:

extram float32_t stalta_cbuff_data[25];

circular_buffer_f32_t stalta_cbuff = {stalta_cbuff_data, 25, 0, 0, true};

stalta_f32_t stalta = {&stalta_cbuff, 5, 15, 10, 0, 0};

The circular buffer structure contains the pointer to the array of data, the length of the array, the index of the begin and end of the buffer, and a boolean indicating if the buffer is empty or not. An improper initialization or usage of these variables could lead to undefined behavior. In MeLa, these are automatically initialized and they are hidden to the developer such that it prevents an improper usage (encapsulation principle). Additionally, the notion of pointer does not exist in MeLa, avoiding errors of writing to unknown memory addresses. For example, forgetting the & operator when defining the *stalta* structure would break the software.

MeLa also hides the mapping of variables that can be stored into the microcontroller SRAM memory (128 kB) or an external SRAM memory that is in a separate chip (8 MB). In the generated C code, each array is mapped to the external memory that has more storage capacity, but small variables are mapped to the internal memory that is faster.

Thus, writing an application with MeLa allows for programmers that are not expert in embedded software programming to write reliable and efficient applications (challenge 1 and 3). Even for an embedded software expert, writing an application with MeLa instead of C reduces the possibilities of making errors.

#### 2.5. Application Verification

##### 2.5.1. Static Analysis of Applications

The static analysis consists of computing properties of the applications from models without executing them. We use it to verify that the applications do not exceed the instrument capacities during their development. In its current version, MeLa is able to compute the processor usage, the battery life time, and the amount of data to transmit by satellite, but other properties, such as memory usage, can be added to the analysis. Each function of the MeLa library is associated with information about execution time used to compute the processor usage. The execution time can be a constant value or an equation with parameters, such as the size of arrays used during the call of the function. Indeed the execution time can change by several orders of magnitude for different sizes of arrays. The functions can also have a specific meaning, such as “this function requests the ascent of the float” or “this function records data to transmit by satellite”, which are interpreted by the analysis tools.

###### Processor Usage

The processor usage of one task *U* is defined by U=C/T, where *C* is the worst-case execution time of the task and *T* is the period of execution of the task. For *n* tasks, the processor usage is $U=\sum_{i=1}^{n}C_{i}/T_{i}$. The worst-case execution time *C* is the longest execution time among the possible execution path of a task (the processing sequence of a continuous acquisition mode is not taken in account since it does not have real time constraints). The execution time of paths is computed with information recorded in the library and size of arrays passed as parameters of functions. The period of execution *T* of a continuous acquisition mode is computed from the sampling frequency of the sensor and the size of the input array, while, for a short acquisition mode, it corresponds to the period that is defined in the Coordinator.

To determine whether the processor is able to execute the tasks in time (i.e., if tasks are schedulables), we use the Liu and Layland utilization bound [21]. This bound defines the maximum processor usage for a set of tasks. A set of *n* tasks is schedulable only if $U\leq n\cdot (2^{1/n}-1)$. It means that a processor can be used at 100% of its capacity for one task (n=1), but only 83% for two tasks (n=2), 76% for four tasks (n=4), and it tends to 69% for an infinite number of tasks. This bound is only valid if (1) tasks have a rate-monotonic priority assignment (as described in Section 2.4.2), (2) tasks have a deadline that is equal to their period of execution, and (3) tasks are independent from each other. These constraints are respected, since (1) the code generation process assigns rate-monotonic priorities to tasks, (2) the deadline corresponds to the arrival period of samples that is the period of execution of tasks, and (3) the functions of the library are written, so that the execution of a task cannot be delayed by another one (i.e., they cannot interfere), or at least the delay must be negligible (e.g., the time to write on the SD card is negligible when compared to the time to switch it on, thus if two tasks write at the time, the writing time is negligible, only the switch on time is taken into account).

###### Duration of the Dive

The duration of a dive is needed in order to estimate the lifetime of the float and the amount of data transmitted each month. It depends on the duration of each stage of the dive. For the surface stage, we consider a constant duration of one hour, even if it can be shorter or longer for a real float, since it depends on the amount of data to transmit by satellite. The descent and ascent stage duration are computed from the depth defined in the mission configuration and an estimated speed of the float. The parking stage has a maximum duration if no ascent request occurs for some time. This maximum duration has to be defined by the developer in the mission configuration. It may be shortened if an application requests the ascent of the float.

To estimate the mean duration of the parking stage, we use a Poisson law that gives the probability to have *k* ascent requests during the default parking stage duration (when considering a fixed probability for ascent requests):

(1) $$p\left(k\right)=\frac{\lambda^{k}}{k!}e^{-\lambda }$$

The λ parameter represents the mean number of ascent requests during the default parking stage duration. It is computed from the mission configuration and the probabilities defined in an application (only probabilities leading to a function that trigger the ascent of the float are used). For example, if the maximum duration of the parking stage is 10 days, and the probability of the ascent is two per week, the λ parameter is equal to 10*2/7=2.86. The probability to have zero ascent request is:

(2) $$p\left(0\right)=e^{-\lambda }$$

Addittionally, the probability to have at least one ascent request is:

(3) $$p\left(k>0\right)=1-e^{-\lambda }$$

The mean parking duration, which is also the mean duration before the first ascent request, corresponds to the mean interval of time between each ascent request (i.e., the invert of the probability defined in the MeLa language) multiplied by the probability to have at least one ascent request during the default parking duration.

**Figure 6:**
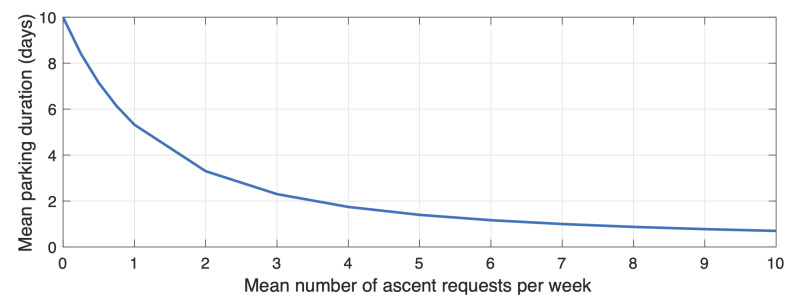
Mean parking duration in function of the mean number of ascent requests for a default parking duration of 10 d.

For example, the probability to have at least one ascent request is $1-e^{-2.86}=0.94$, and the mean parking duration is 7/2*0.94=3.3 d. Figure 6 provides a curve of the mean parking duration as a function of the mean number of ascent requests for a default parking duration of 10 d.

###### Satellite Transmission

The amount of data transmitted by the float are computed from the recording functions called in the application. In the MeLa library, the recording functions are annotated with the amount of data they record for satellite transmission. For functions recording arrays, the amount of data is dependent of the array size passed as a parameter of the function. The probability annotations in the MeLa code are also used for the computation.

###### Battery Life Time

The lifetime of the instrument LT is related to the energy that is contained in the battery $E_{bat}$, the mean energy consumption of a dive $E_{dive}$ and the mean duration of a dive $T_{dive}$:

(4) $$LT=E_{bat}/E_{dive}*T_{dive}$$

The energy contained in the battery (i.e., the battery capacity) is known and the computation of the mean duration of a dive has been introduced previously. The mean energy consumption of a dive is equal to the sum of the energy that is consumed during each of the stages (i.e., descent, parking, ascent, and surface).

For each stage, the energy that is consumed $E_{stage}$ is the sum of the energy consumed by the actuators, the sensors, the acquisition board, and the satellite communication devices. Naturally, when the float is underwater the satellite communication devices are switched off and do not consume any energy.

(5) $$E_{stage}=E_{act}+E_{sens}+E_{board}+E_{com}$$

The energy that is consumed by the actuators $E_{act}$ depends on the mission step. Most of the energy of the descent is consumed by operating a valve at the surface. Once the float is deep enough, the pressure of the water is high enough to transfer oil from the outer bladder to the inner reservoir with almost no energy required from the pump. Thus, we assume that the energy consumption of the descent is a small constant value. For the parking we consider that the energy consumption is null even if there is occasionally some small depth correction. Most of the energy is consumed during the ascent because the pump has to push oil in the outer bladder where the pressure can reach several hundred of bars. We use a quadratic relation between the power consumption and the depth of the float, and add a constant value for the power that is needed to fill the bladder at the surface. The linear relation is a simplified model, we plan to improve it by taking account of the motor efficiency and behavior of the float during the ascent, and then validate it with experimental data.

The energy consumed by the sensors $E_{sens}$ is the product of their activation time with their energy consumption. The activation of a sensor can be intermittent if it is used by a short acquisition mode. Moreover, the same sensor can be used by several applications. Thus, an algorithm computes the activation time of the sensors. For example, if two applications, A and B, use the same sensor, one for two minutes every five minutes and the other for one minute every three minutes, the algorithms compute the pattern of Table 1 that repeats itself every 15 min. Here, the sensor is used nine minutes over a period of 15 min, which is 60% of the time. If the stage during which this sensor is activated has a mean duration of 10 h, the activation time of the sensor is six hours.

The energy consumed by the acquisition board $E_{board}$ is the power consumption of the board multiplied by the mission step duration. This simplified model assumes that the acquisition board power consumption is constant. It is a conservative model, because the sleeping modes, activated when the board does not have any data to process, are not taken into account.

The energy consumed by satellite communication is the number of bytes to transmit $Tx_{bytes}$ divided by the average speed of the transmission $Tx_{speed}$, which gives the duration of the transmission, and multiplied by the power consumption of the transmission device $P_{sat}$:

(6) $$E_{sat}=Tx_{bytes}/Tx_{speed}*P_{sat}$$

## 3. Developing with MeLa

**Figure 7:**
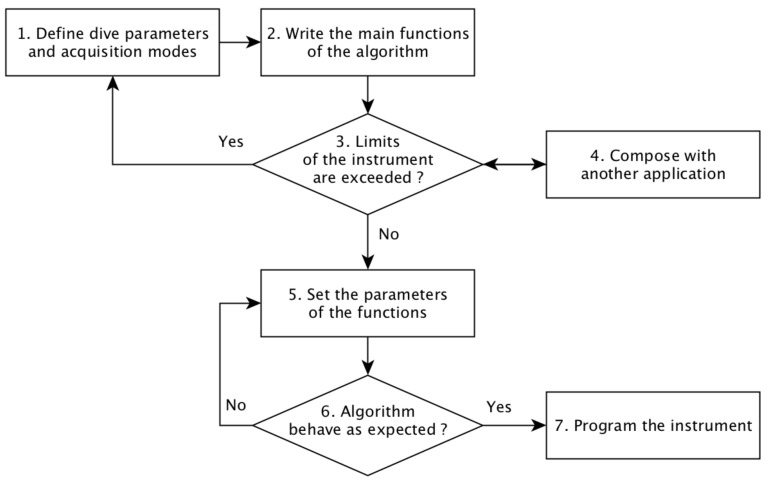
Development workflow with MeLa.

Here, we describe a workflow to develop applications with the MeLa language. Figure 7 illustrates the workflow. The presented workflow allows for verifying that the applications do not exceed the limits of the instrument during the development process, before having a functional application. The programming of the instrument is only done once, at the very end.

The first step is to define the duration and depth of a dive, the acquisition mode, and the coordinator. For each application, the developer has to choose between a continuous or a short acquisition mode. Continuous acquisition modes are more adapted to detect sporadic signals, like those that are emitted by earthquakes, because they process data without stopping. On the other hand, short acquisition modes are more adapted to monitor events that evolve slowly, like temperature or wind, because they have a reduced impact on the battery lifetime of the floats, since the sensor is switched off between each data packet. A good practice for continuous acquisition modes is to have a detection part, with short processing time, in the real-time sequence and a discrimination (classification) part, which often have a long processing time, in a processing sequence. Short acquisition modes only have a processing part, they are not intended for real time detection.

Once the acquisition mode is chosen, the developer defines sampling frequency and writes a first version of the application, with the main functions to be used. The application does not need to be functional in a first step; for example, filter parameters do not yet need to be chosen. Only the information used by models to verify that the limits of the instrument are not exceeded is necessary: the length of the arrays, of the Fourier transforms, the functions used to process the data, and also the probabilities of the conditional branches. The probabilities do not have to be exact, but they should be conservative to obtain a safe estimation of the battery life time and cost of satellite transmission.

If the limits of the instrument are not exceeded, the application can be composed with another application to verify that they will be both able to execute on the same instrument. The dive depth and maximum duration must be the same and, if the same sensor is used at the same time by two applications, the configuration of the sensor must be the same (i.e., sampling frequency). If they are different, an error is raised in order to force the developers to find a compromise. Once they are composed, it is necessary to again verify that the limits of the instrument are not exceeded. Most of the mechanisms used to execute the acquisition modes concurrently are managed by the embedded software code; this includes the scheduling of tasks and the exchange of data from sensors to processing tasks.

The applications can be executed on a laptop (i.e., simulation). Simulation is complementary to static analysis, it focuses on the behavior of the applications instead of the instrument limitations. It allows for a developer to correctly set the parameters of an application without having to program a real instrument, for example, she can verify that an application records as many earthquakes as expected and adjust parameters to improve the performances. Currently, the simulation only handles the processing of data, it does not fully simulate the behavior of the instrument.

The simulation code is generated from the MeLa code and it uses the same library of functions as the one used for the instrument. Most of the function implementations are exactly the same for the simulation and the instrument. It ensures that simulation gives results that are close to those that will be obtained on a float. Some differences exist, because the DSP of the Cortex M4 is not available in a personal laptop. Emulating the float processor would allow to be even closer to the results of the instrument. During the simulation process, the probability values can also be refined.

The code for the instrument is generated from the application. It can be compiled without any modification. Because verification of the limits of the instrument and simulation have been done during the development process, thanks to the MeLa capabilities, the applications can be deployed without requiring additional tests.

## 4. Experiments

We describe two detailed examples of real-life applications in order to illustrate the capabilities of the MeLa language. The first example is the seismic detection algorithm implemented in the original Mermaid floats and the second one is an algorithm that was developed to detect D-calls of blue whales. We specify the algorithms and discuss the results of the analysis and simulation.

### 4.1. Detection of Earthquakes

#### 4.1.1. Scientific Context

Seismic waves that are emitted by earthquakes are used by seismologists to map the interior of the earth. The speed of propagation of seismic waves, especially compressional (P) and shear (S) waves, is dependent on the temperature inside the earth. When an earthquake occurs, measurements of the travel time of the seismic waves allow for imaging cold subducting oceanic lithosphere and hot mantle plumes under volcanic islands, such as Hawaii. In order to have a good image resolution, measurements all around the earth are needed, including in the oceanic regions that represent 70% of the surface of the globe. Scientists have developed the MERMAID floats because of the absence of seismographs in marine areas. These are equipped with a hydrophone (i.e., an underwater microphone) that can observe P and occasionally S waves as acoustic waves transmitted from the ocean floor into the water column. Recently, we have shown how even a small network of Mermaids was able to image a mantle plume beneath the Galapagos Islands [22]. Another experiment is currently underway in the Pacific with 49 Mermaid floats deployed (EarthScope Oceans website: http://geoweb.princeton.edu/people/simons/earthscopeoceans/). The Southern University of Science and Technology will also launch 10 Mermaids in the South China Sea in November 2020 and five in the Indian Ocean next year (Yongshun John Chen, personal communication 2020).

#### 4.1.2. The Seismic Detection Algorithm

The seismic wave detection algorithm is presented in detail in Sukhovich et al. [14]. Here, we give a brief and simplified overview of this algorithm. The first step of the algorithm is the detection of a signal amplitude increase. The signal is filtered by a high pass filter to suppress the micro seismic noise at frequencies below 1 Hz. A Short Term Average over Long Term Average (STA/LTA) is used to detect an elevation of the absolute signal amplitude. In this example the STA/LTA is the mean of the last 10 s of the signal over the mean of the last 100 s. When the result of the STA/LTA exceeds a threshold, it triggers the discrimination part of the algorithm, which decides whether the signal is a seismic wave.

The discrimination algorithm computes the wavelet transform of the signal, equivalent to a bank of six bandpass filters. The six frequency bands are averaged over time and a normalization process is done between the noise part, before the trigger, and the signal part, after the trigger. This leads to a representation of the signal that is similar to a power spectrum, with six values for each frequency band. A criterion is computed from the distance between the measured powers and the center of six reference distributions. The Signal over Noise Ratio (SNR) is also computed. Both values are used in a decision to trigger the recording of the signal and eventually the ascent of the float to obtain a precise position with the GPS at surface.

#### 4.1.3. Implementation with MeLa

The implementation with MeLa requires functions, like STA/LTA, triggers, wavelets transforms, and cumulative distributions. Implementing a specialized algorithm may require the involvement of an embedded software expert to write specific functions in C language in order to add them in the MeLa library. The MeLa language does not offer the full flexibility of a generic programming language in order to ensure the reliability and efficiency of the applications, and to permit the analysis of applications. However, the current library of functions (Table A2) is already generic enough to be of use in many different applications. The MeLa code of the seismic application is accessible on Github, see the Supplementary Materials section.

**Figure 8:**
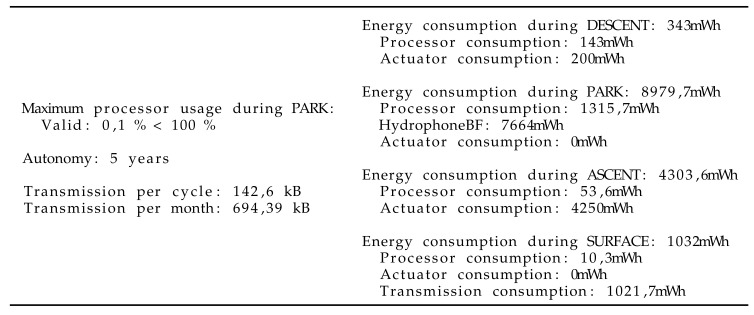
Analysis results given to the developers by MeLa.

Once the seismic application has been implemented with the MeLa language, the analysis tool allows for verifying that the limits of the instrument are not exceeded. Figure 8 shows that the processor is used only 0.1 pct of the time. Indeed the time between each packet of data is long compared to the time required to process them. The autonomy of the float depends on the frequency of the ascent requests; the estimated autonomy was found to be five years if the algorithm records four earthquakes and triggers one ascent per week (2.9 years if it records 10 earthquakes and triggers 10 ascents per week). The estimated amount of data transmitted per month was found to be, respectively, 708 kB and 915 kB. The 708 kB compares well with the Mermaids floats currently operating in the Pacific which transmit 400 kB per month with a compression algorithm that divides the size of data by 2. The processor usage of 0.1 pct is compared to the maximum allowed processor usage that is not necessarily 100 pct if several applications must be executed at the same time (as defined by the Liu and Layland theorem).

We have tested a version of the algorithm that uses floating point numbers instead of integers to filter the micro seismic noise, process the STA/LTA, and compute the wavelet transform. With floating point numbers the processor is used 0.17 pct of the time, higher than the integer implementation but still very low. It shows that choosing a floating point implementation is possible and may be preferable, because it gives flexibility to design the high pass filter that removes micro seismic noise. We have also tried another version of the algorithm that is based on a Fourier transform instead of the numeric filter. It was found early that using a Fourier transform (1024 samples processed every 512 samples) increases the processor usage to 0.7 pct. This demonstrates that using Fourier transforms in real time for seismic monitoring is possible, but it will use more processor time than the original algorithm.

#### 4.1.4. Evaluation of the Algorithm

The algorithm has been tested on a laptop with the simulation code that was generated from the MeLa language. To feed the algorithm, we have used 10 months of continuous recording from a Mermaid recovered in August 2019. We compared the results of the simulation with the events sent through satellite by the Mermaid before its recovery. All 12 earthquakes that were detected by the Mermaid are also detected by the algorithm implemented with the MeLa language. Three additional non seismic events have also been detected with the MeLa language, indicating slight differences in the implementations. Nevertheless, we conclude that the MeLa language can be used to implement advanced algorithms.

### 4.2. Detection of Blue Whales

#### 4.2.1. Scientific Context

Whales have become an important topic of study among marine biologists and scientists, as they play a very crucial role in the health of the ocean ecosystem. They participate in the food chain by absorbing krill [23] and help to capture carbon from the atmosphere by rejecting nutrients that stimulate the growth of phytoplankton [24]. Despite these important roles, whales are endangered worldwide. In particular, during the 20th century, the blue whale was an important whaling target. Nowadays, like other large whales, blue whales are threatened by other human activities (e.g., climate change impact on krill, ship strikes, fishing gears, toxic substances). The best solution for the protection and conservation involves a better understanding of their spatial distribution, migration, of their social structure, and how they communicate with one another. Long term acoustic monitoring at a global scale would help those studies. Processing, such as counting the whale calls [25], must be done on the instrument, because of the limited satellite transmission bandwidth.

#### 4.2.2. The Blue Whale Detection Algorithm

**Figure 9:**
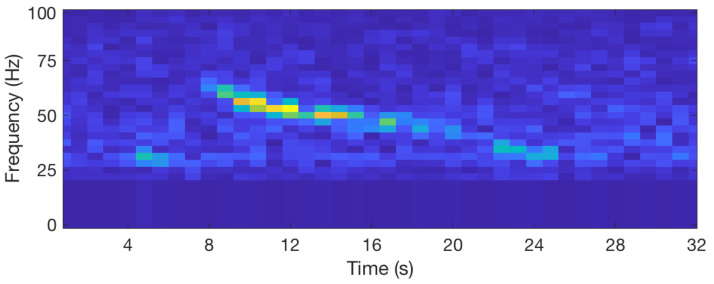
Spectrogram of a blue whale D-call with low frequencies removed.

**Figure 10:**
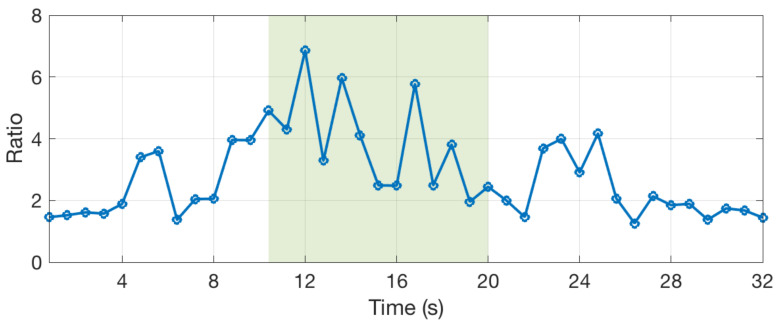
Ratio of spectrum amplitudes for each spectrogram window.

**Figure 11:**
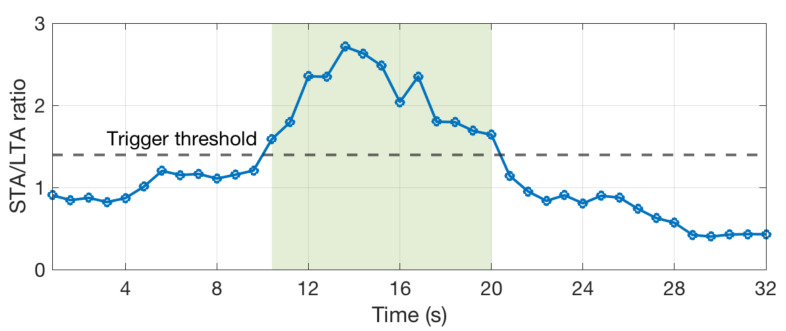
Short Term Average over Long Term Average (STA/LTA) computed from the ratio Figure 10.

**Figure 12:**
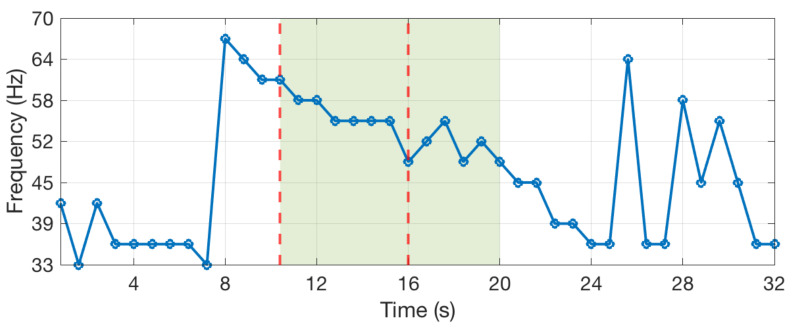
Frequencies corresponding to the maximum amplitudes of the spectrogram.

Blue whales emit different sounds, called A, B, C, and D calls, which are involved in their social behaviors [26]. We have developed an algorithm that detects the occurrence of D-calls and records the occurrence dates. The spectrogram of D-calls has a very specific shape. This is a narrow band signal that sweeps typically from 80 to 20 Hz, as shown in Figure 9. The algorithm detects this shape in real time. First, the algorithm computes the spectrum *S* of the signal with a Fourier transform of 64 samples and a window overlap of 50% (32 samples). After removing noise at low frequencies, below 20 Hz, the algorithm searches for the six highest values of the spectrum and computes a ratio between the two highest over the fifth and sixth highest (max1(S)+max2(S))/(max5(S)+max6(S)). This is done for successive time windows resulting in the curve that is shown in Figure 10. The computed ratio is only high when a signal with a narrow frequency band exists. However, the ratio is not always very stable, especially if the signal is weak. Therefore, we compute the STA/LTA average that smoothes the curve, as shown in Figure 11. If the value of the STA/LTA exceeds a trigger value, then the ratio is put inside a buffer to be used in the next step of the algorithm. The frequency at which is found the maximum amplitude of the spectra (Figure 12) is also kept in memory.

After the value of the STA/LTA drops under the trigger threshold, the two curves are used to discriminate D-calls from other noises. Only the part of the curves between the trigger and detrigger (green highlight) are in memory at this time. In order to remove potentially wrong values at the end or at the beginning of the curve, the time window is truncated. We use the frequency curve on Figure 12 and only select the part between the maximum and minimum value (first minimum), as shown with the two dashed red lines. Subsequently, we count the number of times the frequency changes downward, upward, or keeps the same value (the frequency can only take 32 values corresponding to the frequency bins of the Fourier transform). For the highlighted part of the Figure 12, the frequency goes downward three times and keep the same values five times. Finally, several checks are done in order to validate that the signal corresponds to a blue whale D-call and, if these are all valid, the date of the detection is recorded. The checks have been defined empirically and are the following:

- The length of the detection must be above 4 successive windows (yellow part of Figure 11).
- The mean value of the ratio must be above 2.5.
- The number of times the frequency goes downward between two successive windows must be more than 3 times the number of times the frequency goes upward.
- The number of times the frequency goes downward must be more than 2.
- The number of times the frequency goes downward must be more than 0.25 times the number of times the frequency stays stable.
- The maximum frequency must be above 40 Hz.
- The maximum frequency must not change by more than 20 Hz between within two points (0.8 s).

The algorithm could be improved by keeping values before the trigger that is a little late when compared to the D-call arrival. Moreover, the discrimination process could be optimized with machine learning and input, such as the features listed above or the ratio (Figure 10) and frequency curves (Figure 12).

#### 4.2.3. Implementation with MeLa

The algorithm has been implemented with the MeLa language. The code is accessible on Github, see the Supplementary Materials section. For this application, the analysis estimates a processor usage of 2 pct. The autonomy of the float is found to be 4.6 years; this is less than the seismic detection application because the power consumption of the sensor is higher, due to a higher sampling frequency. The estimated amount of data transmitted each month is only 14 kB. This is much less than the seismic application, because only timestamps are sent through satellite communication. However, the probability to record a blue whales D-call is estimated to be much higher with five records per hour. If 20 s of sounds where recorded for each detection, the amount of data to transmit each month would be 56,047 kB. Transmitting such an amount of data by satellite increases the costs of satellite transmission and reduces the life time of the float to 0.7 years.

Once a first version of the algorithm is ready we can compose it with the seismic application. A first error appears because the maximum duration of the dive and depth are not equal for the two applications, thus we defined both to 10 d and 1500 m depth. Subsequently, another error appears because the two applications use the same sensor, but at different sampling frequency. A solution could be to use decimation, but this has not been implemented in the language. Alternatively, we could adapt the seismic algorithm to support higher sampling frequency. Instead, we decided to use the two outputs of the hydrophone, one for low frequencies and one for high frequencies. However using the two outputs of the hydrophone has a noticeable effect on the power consumption. Instead of an autonomy of 5 and 4.6 years for the two separated applications, MeLa computes an autonomy of 2.8 years if the two applications are composed, to be installed on the same instrument. The processor usage is still very low (i.e., 2%), because neither applications require a lot of processing time.

At this point, the algorithm has not been finalized, since several coefficients still have to be defined or refined. For example, the STA/LTA length or the checks done in order to validate that the signal is blue whale D-call. Simulating the algorithm on a personal laptop with experimental data allowed finalizing the algorithm without programming a real instrument. The finalized algorithm is the one described in the precedent section.

#### 4.2.4. Evaluation of the Algorithm

Contrary to the seismic detection algorithm, the blue whales detection algorithm has never been evaluated before. Thus, we compare its performances with state-of-the-art algorithms and datasets from the Detection, Classification, Localization, Density Estimation (DCLDE) community.

##### Evaluation Protocol

We used the data from the DCLDE 2015 challenge that has been recorded with High-frequency Acoustic Recordings Packages deployed off the southern and central coast of California. The data spans all four seasons over the 2009–2013 period (See the full dataset documentation at http://cetus.ucsd.edu/dclde/datasetDocumentation.html.) but we used a 50h-long subset that has been annotated during a recent collaborative campaign [27]. The annotators have identified a total of 916 D-calls, plus 101 40 Hz annotated sound events. The high-frequency data have been decimated to 200 Hz bandwidth to feed the MeLa algorithm.

Furthermore, we used as performance metrics the Precision (i.e. total number of detected calls) and Recall (i.e. total number of annotated calls), while using the python library *sed_eval* (https://tut-arg.github.io/sed_eval/sound_event.html) [28] for their implementation. As we are interested in soft detection of sound events, i.e. without the estimation of D-calls duration, we only used the onset time in our evaluation metrics with a large time collar of 6s from the reference time onset, within which the estimated onset needs to fit in, so that a detection is counted as being correct.

The detection performance of the MeLa algorithm was compared to a custom Convolutional Neural Network (CNN)-based model. CNNs are increasingly used in classification applications that involve acoustics [29,30,31] and have recently revealed promising performance for marine mammal detection [32,33,34,35]. We used the ResNet architecture, which is a deep neural network using skip connections or short-cuts to jump over some layers [36]. Only 18 layers were stacked to avoid overfitting, as the training set is not very large. It was trained from scratch to handle the size of the mel-spectrogram [37] images (110 × 90 instead of the initial shape of 224 × 224). Each image is generated from 5s audio excerpts sequentially taken from the recordings. Our implemented version is based on existing open source codes (https://github.com/keras-team/keras-contrib/blob/master/keras_contrib/applications/resnet.py).

##### Results

The Resnet model has a precision of 82% and recall of 69% for 765 overall detected events. The MeLa algorithm has a precision of 99% and recall of 55% for 513 overall detected events. Even if the recall is lower for the MeLa algorithm, its precision is better, which is an advantage if the algorithm is used in an alarm system to prevent ship collision. The main differences between the algorithms reside in the resources that they use:

- For the execution time, it takes 12 s for Resnet to process a five minutes long recording whereas it takes only 15 milliseconds for the algorithm written in MeLa; for one year of data, it is 14 d against 26 min. Moreover the CNN has been executed on GPU Geforce GTX1060, whereas the MeLa algorithm has been executed on a laptop.
- The network Resnet size is 134.4 MB whereas the programmable memory of the float has only 256 kB of space, the MeLa algorithm size is 410 kB if compiled for a laptop and 139 kB if compiled for the float (and 148 kB if compiled with the seismic detection algorithm).
- The GPU used in the evaluation has a power consumption of 116 Watts, which would consume the 4 kWatts.h of energy that is available on the MERMAID float just after a day and a half. If the MeLa algorithm is used on the GPU to process one year of data, it will consume 46 W.h instead of 39 kW.h for the CNN; this is equivalent to the energy that is required by an electric car to travel, respectively, 300 m and 260 km.

The algorithms that were developed with MeLa are suited to program Mermaid floats, but can also be used to process large amounts of data with low execution time and energy consumption, which also mean less environmental impact.

## 5. Discussion and Conclusions

We have developed a programming language, called MeLa, dedicated to the Mermaid instrument, a multidisciplinary float that can monitor the oceanic environment with multiple sensors. The language is a DSL created using a Model Driven Engineering (MDE) approach [38,39,40]. The language allows for scientists that are not specialists of embedded systems to write reliable and efficient applications of the Mermaid instrument. It uses models to verify that the applications comply with the limited resources of the instrument and to compose (i.e., combine) applications developed independently, but to deploy in a same instrument. The code to execute the applications on a personal computer and the code for the instrument are generated from models using rules that were defined by embedded software experts.

Generic programming languages, such as C, Java or Python, do not have functionalities, such as those offered by MeLa. When compared to MeLa, writing applications with those languages increases the risk of making errors that may compromise the integrity of the instrument, even for an embedded software expert. A software library or a framework, such as Arduino (https://www.arduino.cc/), can reduce the risks with specialized functions that encapsulate low-level concerns (e.g., a function that read a sensor) and by defining a default architecture for the code (e.g., with a setup and a loop function for Arduino). It helps the developers, but the code is still written with a generic programming language for which the risk of errors is higher. Furthermore, those languages do not incorporate analysis capabilities that are included in MeLa, because it requires platform-specific information (e.g., energy consumption, execution time) that are not compatible with their generic aspect. Analysis tools exist, but also need platform-specific information, for example, the processor speed or the time that is required to read a sensor.

There exists a few DSLs that are comparable to MeLa, such as CPAL [41], MAUVE [42], or Mbeddr [43]. However they have mostly been designed for embedded software developers. They offer high-level programming abstractions but are still too close to embedded software concerns; for example, in CPAL and MeLa the processor usage is computed from execution times that must be manually inserted into the code, whereas, for MeLa, this information is hidden in the library of functions. Moreover, they do not include a composition tool for combining several applications.

MeLa has been developed for the Mermaid float, but it could also be used to program other instruments. One of those could be the AudioMoth [44,45,46], an autonomous acoustic monitoring device that can be programmed with applications, for example, to detect cicadas or bat calls. More generally, the language could be used for most of the sensors used in the Internet of Things [47,48]. However, it is presently limited to sensors and cannot be used to program actuators or display devices.

Mela is also limited to program instruments that exist, for which a model of operation can be created and measurements, such as execution time or energy, can be done. The development of embedded systems from scratch is not possible with MeLa; it would require many other features, such as adding a large library of configurable software and hardware components to create a system, and also taking account of physical considerations (e.g., pressure change, and its effect on actuators power consumption for Mermaid).

Industry, such as automotive or spatial industry, have budgets to follow very strict development processes with international standards, quality insurance, static analysis, and the like [49]. Such processes cannot be followed each time that a scientist wants to add an application to the Mermaid, it would require budgets that many scientists do not have. Thus, instead of following the classical development process that consists of developing a signal processing application in a language such as Matlab (https://fr.mathworks.com/), refine it into embedded software code, integrate it in an embedded software architecture, and test it on the embedded hardware, our approach allows for developing applications in a single step, and by a non-specialist. Such an approach could also be used for other multidisciplinary instruments that require adaptability.

We plan to integrate several other sensors (e.g., chemical, magnetic, optical) to the Mermaid instrument. Because the instrument can be deployed for several years (depending on the applications), we plan to add over the air programming capabilities in order to enable scientists to modify the software after deployment. The MeLa language itself can be improved with several features such as:

- Raising its level of abstraction, for example, by allowing the developers to define an overlap between each packet of data that can be useful for the computation of spectrograms.
- Adding machine learning capabilities to automate, at least in part, the development of algorithms. Models, such as decision trees or neural networks, can be integrated and trained into an application. It is also possible to optimize the value of specific parameters with linear regression, for example, a threshold in a condition.
- Computing RAM, flash, and programmable memory usage that are very limited in embedded systems. It would help to prevent exceed of their capacity.
- Applications using the same sensor at different sampling frequencies should be able to be composed using a decimation filter. Such a filter must be steep enough to prevent aliasing and doing it automatically is challenging.
- Development tools to offer a better experience to developers and incite them to adopt the language. For example, a development environment with auto completion and highlighting of the code snippets that use the most of resources, or plotting functions for simulation.

We expect that MeLa, certainly after adding such features, will stimulate the creation of applications from multiple disciplines and it will lead to significant cost savings for future programs to monitor the oceanic environment.

## Figures and Tables

**Table 1:** Activation pattern of a sensor used by two applications allowing to compute the energy consumption of the sensor. The first row is the time, the three other rows are the activation state of the sensor, with 1 for the activated state and 0 for the disabled state.

Time in minutes	1	2	3	4	5	6	7	8	9	10	11	12	13	14	15	...
Sensor used by app A	1	1	0	0	0	1	1	0	0	0	1	1	0	0	0	...
Sensor used by app B	1	0	0	1	0	0	1	0	0	1	0	0	1	0	0	...
Total sensor usage	1	1	0	1	0	1	1	0	0	1	1	1	1	0	0	...

## Supplementary Materials

Source code of the MeLa language and example of applications are available at: https://github.com/sebastienbx/MeLa_Sensors2020.

## Abbreviations

The following abbreviations are used in this manuscript:

Mermaid	Mobile Earthquake Recording Device in Marine Areas by Independent Divers
MeLa	Mermaid Language
OBS	Ocean Bottom Seismometers
UPA	Underwater Passive Acoustic
CTD	Conductivity Temperature Depth
GPS	Global Positioning System
RUDICS	Router-Based Unrestricted Digital Internetworking Connectivity Solutions
SRAM	Static Random Access Memory
DSP	Digital Signal Processor
RTOS	Real Time Operating System
DCLDE	Detection, Classification, Localization, Density Estimation
STA/LTA	Short Term Average over Long Term Average
CNN	Convolutional Neural Network
DSL	Domain Specific Language
MDE	Model Driven Engineering

## Appendix A

**Table A1:** List of data types available in the current version of MeLa.

*# Values* Bool, Int, Float ComplexInt, ComplexFloat String # *Arrays* ArrayInt, ArrayFloat ArrayComplexInt, ArrayComplexFloat # *Buffers* BufferInt, BufferFloat # *Specific types for processing* FFTInt, FFTFloat, IIRInt, IIRFloat, FIRInt, FIRFloat, CDF24Int, CDF24Float, StaLtaInt, StaLtaFloat, TriggerInt, TriggerFloat, DistributionInt, DistributionFloat # *Utils* File

**Table A2:** List of functions available in the current version of MeLa.

# *Array functions* put(); Put a value in an array get(); Get a value from an array copy(); Copy an array to another array toComplex(); Convert an integer or a **float** to a complex number toFloat(); Convert an integer to a **float** toInt(); Convert a **float** to an integer select(); Select a portion of an array to work on unselect(); Work on the whole array clear(); Delete all values in the array # *Buffer functions* push(); Add a value to the end of the buffer toArray(); Copy the content of the buffer to an array # *Processing functions* fft(); Compute a Fourier transform cdf24(); Compute a CDF24 wavelet transform cdf24ScalesPower(); Compute the power of each scale of a CDF24 iir(); Infinite impulse response filter fir(); Finite impulse response filter stalta(); Short term over **long** term average trigger(); Return true **for** high or low value, or rising or falling edge, compared to a threshold cumulativeDistribution(); Compute the cumulative distribution max(); Find the maximum value and its index in an array min(); Find the minimum value and its index in an array mean(); Compute the mean value of an array sum(); Sum all the elements of an array energy(); Compute the energy, defined as the sum of the squared values of an array rms(); Root means square stdDev(); Standard deviation var(); Variance abs(); Absolute value add(); Add elements of an array with a value or add two arrays sub(); Subtract elements of an array with a value or subtract of two arrays mult(); Multiply elements of an array with a value or divide two arrays div(); Divide elements of an array with a value or divide two arrays diff (); Forward finite difference (derivative approximation) dotProduct(); Dot product of two arrays negate(); Negates each value of an array conv(); Convolution between two array corr(); Correlation between two array sqrt(); Square root cos(); Cosinus sin(); Sinus log10(); Common logarithm pow(); Power of n magnitude(); Magnitude of a complex number # *Utility functions* ascentRequest(); Request the ascent of the **float** getTimestamp(); Get the current date with a resolution of 1 second record(); Record a value, an array, a buffer or a string in a file getSampleIndex(); Only **for** simulation. Get the index of current sample read in the input file
